# miR-27a inhibits cervical adenocarcinoma progression by downregulating the TGF-βRI signaling pathway

**DOI:** 10.1038/s41419-018-0431-2

**Published:** 2018-03-12

**Authors:** Fang Fang, Bangxing Huang, Si Sun, Man Xiao, Jing Guo, Xiaoqing Yi, Jing Cai, Zehua Wang

**Affiliations:** 1 Department of Gynecology and Obstetrics, Union Hospital, Tongji Medical College, Huazhong University of Science and Technology, Wuhan, 430022 China; 2 Department of Pathology, Union Hospital, Tongji Medical College, Huazhong University of Science and Technology, Wuhan, 430022 China

## Abstract

High-risk human papillomavirus infection is essential for the malignant transformation of cervical cancer and can inhibit host miR-27a expression. We investigated the role and mechanism of miR-27a in cervical cancer progression. miR-27a is decreased in cervical cancer cell lines and miR-27a-agomir inhibited the cell proliferation, migration, and invasion properties of HeLa (adenocarcinoma) cells, but not in SiHa cells (squamous cell carcinoma). Luciferase assays revealed that miR-27a directly targets the 3′-UTR of transforming growth factor beta receptor I (TGF-βRI) and downregulates TGF-β signaling. The co-transfection of a TGF-βRI expression vector largely restored the inhibition of TGF-β signaling, cell proliferation, migration, and invasion mediated by miR-27a-agomir. Also, miR-27a-agomir slows down the growth of subcutaneous HeLa xenografts and downregulates the TGF-βRI expression and TGF-β signaling in tumor in vivo. Tissue microarray analysis revealed a low miR-27a level in adenocarcinoma cells, but not in squamous cell carcinoma cells, which was negatively associated with TGF-βRI expression. High TGF-βRI correlated with deep stromal invasion and lymph node metastasis. These results suggest that miR-27a acts as a tumor suppressor in cervical cancer, especially in adenocarcinoma, by inhibiting TGF-βRI signaling pathway. Thus, enhancing miR-27a expression and function may be a novel treatment strategy for cervical adenocarcinoma.

## Introduction

Worldwide, 70% of cervical cancer cases occur in less developed countries; in these regions, cervical cancer is often diagnosed at an advanced stage and is the leading cause of cancer-related deaths among women^1^. Persistent, high-risk human papillomavirus (HPV) infection is essential for the malignant transformation of cervical cancer. However, the molecular mechanism underlying cervical cancer progression is not fully understood.

MicroRNAs (miRNAs) are a class of small non-coding RNA molecules (20–24 nucleotides) that suppress gene expression by binding to the 3′-untranslated region (3′-UTR) of target mRNAs and subsequently either induce mRNA degradation or inhibit translation^2^. miRNAs play essential roles in the initiation and progression of human cancers^3^. Emerging evidence suggests that host and viral miRNAs are involved in virus-associated cancers, such as hepatocellular carcinoma and lymphomas^4–6^. In cervical cancer and cervical intraepithelial neoplasia, a variety of miRNAs were found to be affected by oncogenic HPV infection^7^. Among these miRNAs, several have been proposed to promote the malignant transformation and progression of cervical cancer. For example, E6/E7 oncoproteins of HPV 16 mediated miR-184 reduction and miR-27b increase that contributed to the accelerated proliferation of cervical cancer cells^8,9^. miR-27a was also downregulated in cervical cancer by HPV16 and HPV18 infection^10^. However, the role of miR-27a in cervical cancer progression remains largely unknown. Here, we aimed to clarify the effects of miR-27a on cervical cancer cell malignant properties and the underlying molecular mechanism.

## Results

### miR-27a inhibits cervical cancer cell proliferation, survival, and invasion

**Fig. 1:**
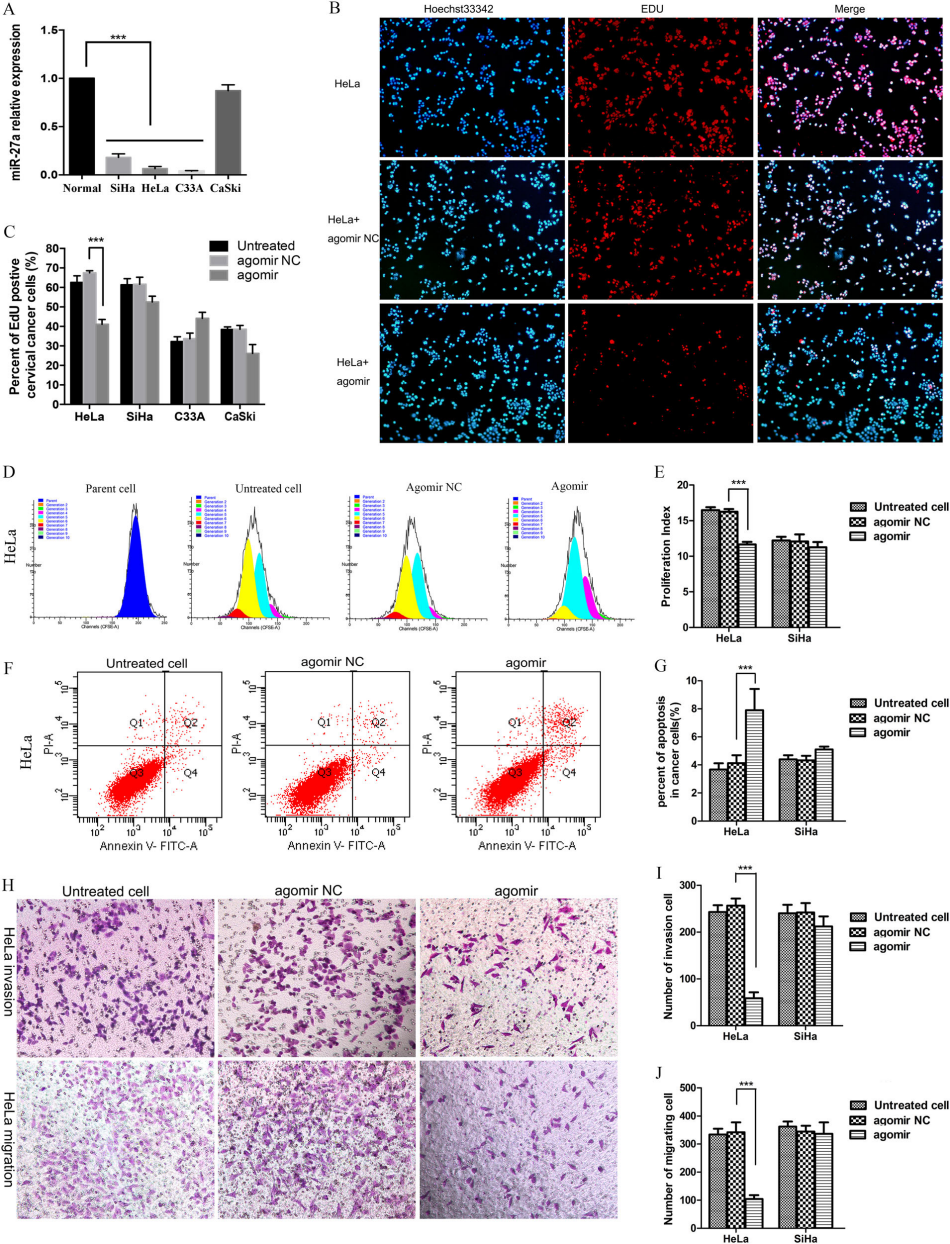
miR-27a inhibits cervical cancer cell proliferation, migration, and invasion and promotes cell apoptosis. **a** qRT-PCR for miR-27a mRNA with U6 as a reference. miR-27a is downregulated in cervical cancer cell lines HeLa, SiHa, and C33A compared to normal cervical cells. **b** Cells were treated with miR-27a agomir. Untreated cells and cells transfected with agomir NC served as controls. Representative images of EdU incorporation assays of HeLa cells. Proliferating nucleus were labeled with EdU (red) while total nuclei were counterstained with Hoechst33342 (blue). **c** Quantitative analysis of the results of EdU assays of HeLa, SiHa, C33A, and CaSki cells. **d** Representative images of flow cytometry analysis of CFSE-positive HeLa cells show the cell fractions of sequential generations (depicted by the different colors). **e** The cell proliferation indexes were calculated according to the CFSE flow cytometry analysis results. The histogram shows the proliferation index in HeLa and SiHa cells transfected with miR-27a agomir versus the control groups. **f** Representative images of flow cytometry apoptosis assays of HeLa cells. **g** The histogram shows the quantification of apoptosis assay results in HeLa and SiHa cells. Representative images of invasion and migration assays of HeLa cells. **I**–**J** The histograms show the number of invading and migrating cervical cancer cells. ***P < 0.001

The miR-27a expression level in the cervical cancer cell lines HeLa, SiHa, and C33A was significantly decreased compared with normal cervical epithelia, especially in the cervical adenocarcinoma (CADC) cell lines HeLa (HPV-18 positive) and C33A (HPV negative, gastric type adenocarcinoma), but not in CaSki cervical squamous cell carcinoma (CSCC) (Fig. 1a). To understand the functional significance of the abrogated miR-27a expression in tumor cells, we transfected control and miR-27a-agomir into cervical cancer cells and monitored changes in cell proliferation, apoptosis, migration, and invasion. Agomirs are chemically-modified double-strand miRNA mimics, which can efficiently mimic mature endogenous miRNAs after transfection into cells. In HeLa cells, miR-27a-agomir markedly reduced EdU-positive proliferating cells compared with the control (Fig. 1b, c). The proliferation index derived from flow cytometry analysis further confirmed the reduction of cell proliferation in the HeLa cells transfected with miR-27a-agomir (Fig. 1d, e, supplemental Fig. S1A). In addition, miR-27a-agomir dramatically increased the apoptotic cell population and inhibited the migration and invasion abilities in HeLa cells (Fig. 1f–j). However, the inhibitory effects of miR-27a on cell proliferation, survival, migration, and invasion were not observed in SiHa, C33A, or CaSKi cells (Fig. 1c–j, supplemental Fig. S1A-F).

### miR-27a regulates TGF-βRI expression and TGF-β signaling

**Fig. 2:**
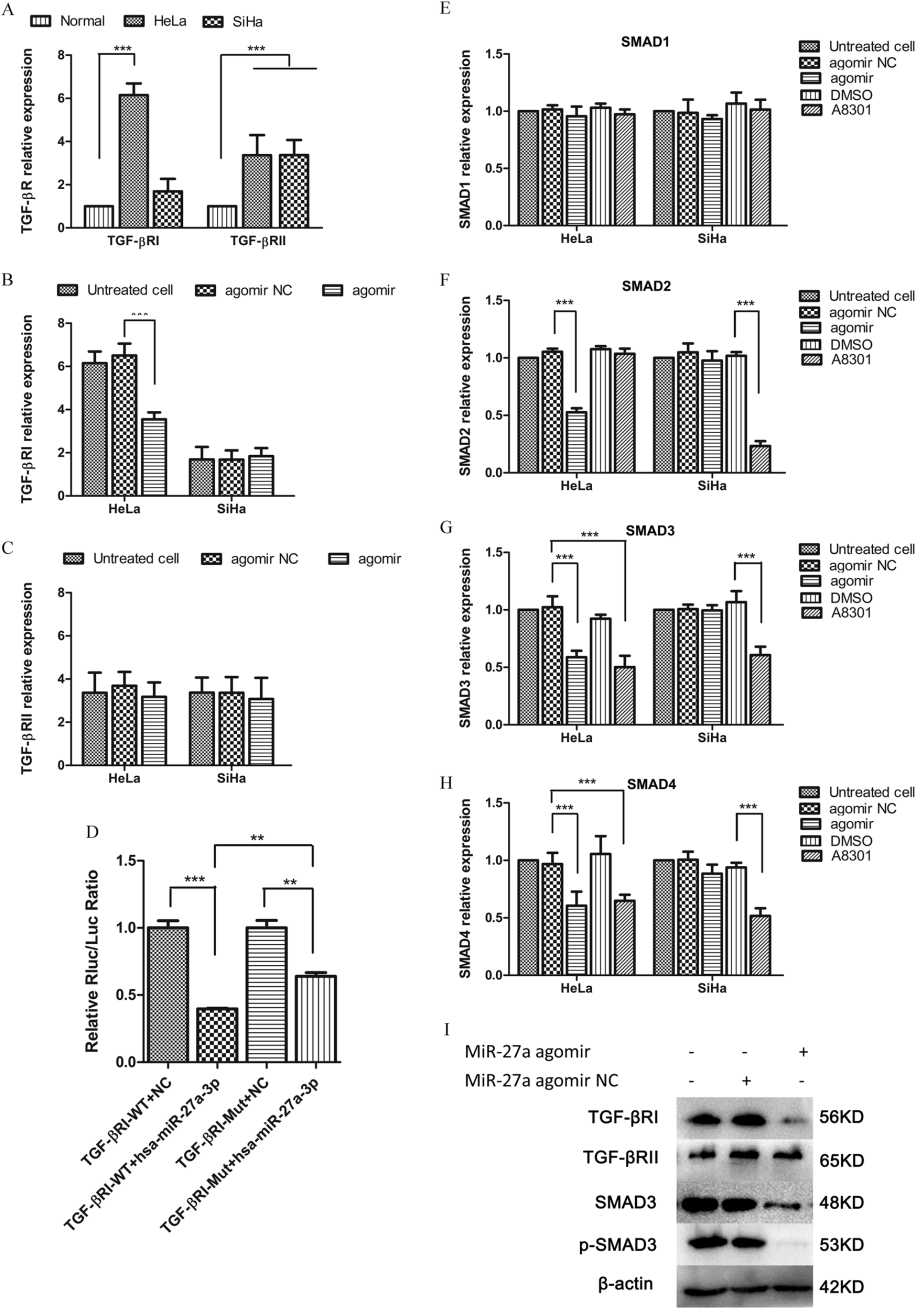
miR-27a directly targets TGF-βRI and represses the TGF-β signaling. **a** qRT-PCR analysis of TGF-βRI and TGF-βRII expression in cervical cancer cell lines versus normal cervical epithelia. **b** qRT-PCR. TGF-βRI expression was downregulated by miR-27a agomir in HeLa, but not in SiHa. **c** qRT-PCR. TGF-βRII levels in three cervical cancer cell lines were not affected by agomir transfection. **d** Results of double-luciferase reporter assays show the luciferase activity of the wild type TGF-βRI 3′-UTR (TGF-βRI-WT) and the mutant one (TGF-βRI-Mut) in the absence or presence of hsp-miR-27a-3p mimics. **e**–**h** qRT-PCR. Expression levels of TGF-βRI, SMAD2, SMAD3, and SMAD4 in cervical cancer cells that were transfected with agomir NC or miR-27a agomir. **i** Western blot. TGF-βRI, SMAD3, and p-SMAD3 were downregulated by miR-27a agomir transfection. **P < 0.01; ***, *P* < 0.001

To elucidate the underlying molecular mechanism of miR-27a regulation, we predicted the potential targets of miR-27a using GeneGO analysis. Data mining and pathway analyses showed that TGF-βRI was a potential target of miR-27a (Supplementary Fig. S2). To test this hypothesis, we first compared the mRNA levels of TGF-βRI and TGF-βRII in normal human cervical epithelia with that in cervical cancer cell lines. Inversely correlating with miR-27a expression, the TGF-βRI level was notably higher in HeLa cells, but not in SiHa cells, compared with normal human cervical epithelia. The TGF-βRII mRNA level was increased in both cervical cancer cell lines (Fig. 2a). More importantly, miR-27a-agomir significantly reduced the TGF-βRI level but not the TGF-βRII level in HeLa cells, suggesting that miR-27a specifically targets TGF-βRI rather than TGF-βRII (Fig. 2b, c). In SiHa cells, however, miR-27a-agomir did not significantly affect the TGF-βRI and TGF-βRII level (Fig. 2b, c). In addition, we treated cells with miR-27a-3p antagomir and miR-27a-5p antagomir. The miR-27a-3p antagomir, but not the miR-27a-5p antagomir, increased the TGF-βRI mRNA level in HeLa cells (Supplemental Fig. S3A), which indicated that miR-27a might interact with TGF-βRI through its 3p mature form.

To determine whether miR-27a directly targets the 3′-UTR of TGF-βRI mRNA, we attached this 3′-UTR to Renilla luciferase (RL) reporter genes and expressed the resulting reporter with or without miR-27a mimics in 293 T cells. As shown in Fig. 2d, luciferase activity was repressed robustly in the cells co-transfected with miR-27a mimics. We generated a mutation at the predicted miR-27a targeting site in the 3′-UTR of TGFβ-RI mRNA and applied it to the same luciferase assay (Supplemental Fig. S3B). Compared with the wild-type reporter, the miR-27a-mediated inhibitory effect on luciferase activity was greatly attenuated when the mutated reporter was co-transfected with miR-27a mimics. The inhibition of TGF-βR1 by miR-27a mimics was not completely abrogated by the mutation we performed, which may be due to unpredicted miRNA binding sites in the 3′-UTR of TGF-βRI mRNA. Therefore, these results together allow us to conclude that miR-27a specifically targets the 3′-UTR of TGF-βRI mRNA and inhibits its gene expression.

SMADs, homologues of the Drosophila 'mothers against decapentaplegic', are key signal inducers during TGF-β signaling^11^. qRT-PCR analyses revealed that miR-27a-agomir transfection significantly decreased SMAD2, SMAD3, and SMAD4, but not SMAD1, mRNA levels in HeLa cells. However, the SMAD expression in SiHa cells was not affected by the miR-27a-agomir. Treatment with A8301, a TGF-βRI kinase activity inhibitor, also reduced SMAD3 and SMAD4 expression in HeLa cells (Fig. 2e, h). In C33A cells, both miR-27a agomir and A8301 decreased SMAD2 (Supplemental Fig. S3C). We further used two TGF-βRI specific siRNAs to knockdown TGF-βRI in HeLa, which also leaded to decreases in SMAD2 and SMAD3 mRNA accompanied with suppressed cell proliferation (Supplemental Fig. S4 A-E). Additionally, the inhibition of TGF-βRI and SMAD3 expression and SMAD3 phosphorylation by miR-27a-agomir transfection was confirmed by western blot assays (Fig. 2i).

### miR-27a functions through targeting TGF-βRI

**Fig. 3:**
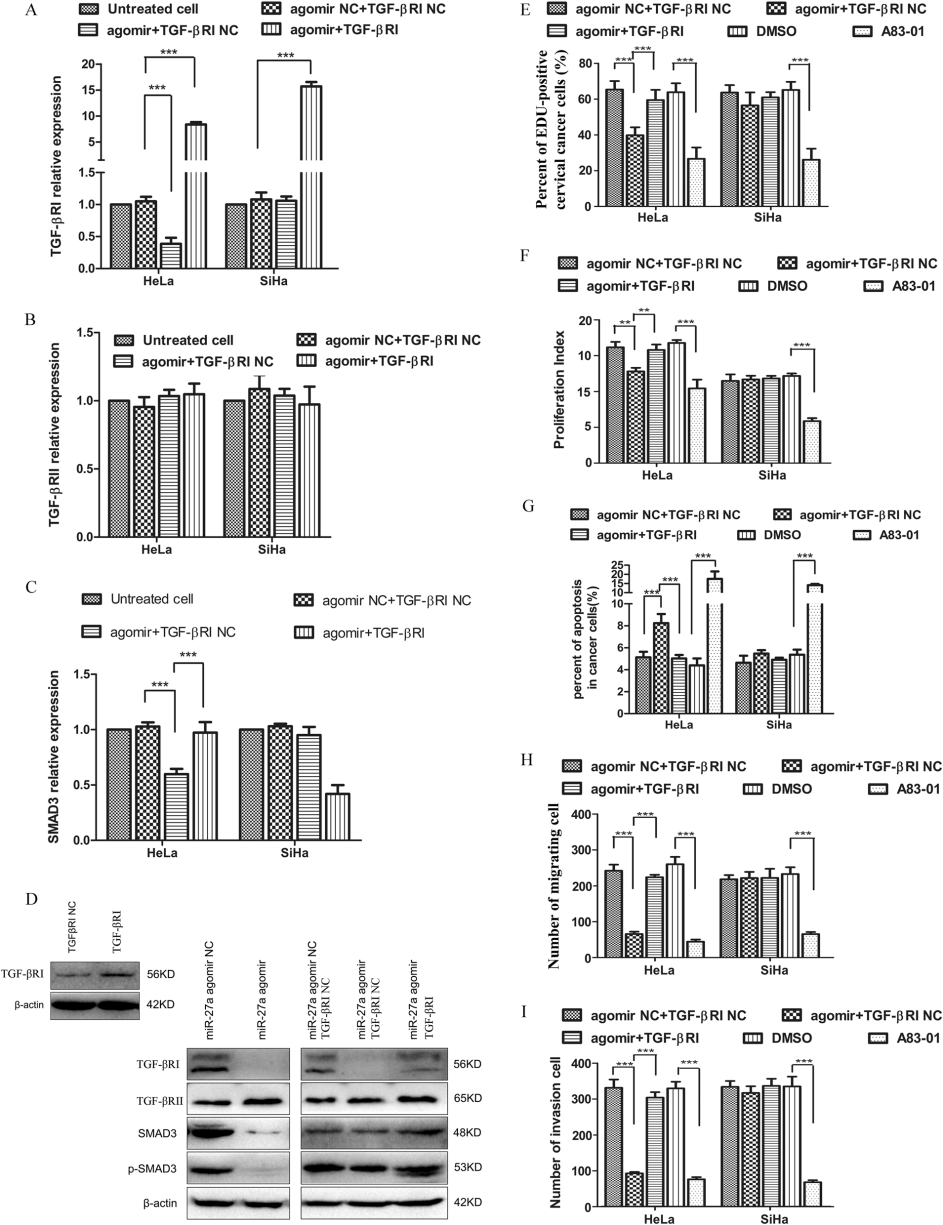
miR-27a functions as a tumor suppressor through repressing TGF-βRI expression and TGF-β signaling. **a**–**c** qRT-PCR analysis of TGF-βRI, TGF-βRII, and SMAD3 expression in cervical cancer cells that were transfected with miR-27a agomir in combination with TGF-βRI expression vector or control vector. **d** Western blot analysis of TGF-βRI, TGF-βRII, SMAD3, and p-SMAD3 levels in cervical cancer cells that were transfected with miR-27a agomir in combination with TGF-βRI expression vector or control vector. A83-01, a TGF-β signaling inhibitor, serves as positive control. DMSO is solvent control of A83-01 treatment. **e**–**i** The co-transfection of miR-27a agomir and TGF-βRI expression vector relieves the tumor inhibition effects mediated by miR-27a agomir. **e** EdU assays. **f** CFSE proliferation assays. **g** Invasion assays. **h** Migration assays. **i** Flow cytometry apoptosis assays. ***P < 0.001

Having demonstrated that miR-27a directly targets TGF-βRI and inhibits the TGF-β signaling pathway, we determined whether the miR-27a-agomir-mediated phenotypic changes of cervical cancer cells resulted from the suppressed TGF-β signaling. To test this possibility, we transfected cervical cancer cells with both the miR-27a-agomir and TGF-βRI expression vector. The downregulated TGF-βRI expression by miR-27a-agomir could be reactivated by the TGF-βRI expression vector in HeLa cells, whereas TGF-βRII expression was not affected (Fig. 3a, b). We further examined SMAD3 expression and phosphorylation in these cells because SMAD3 represents the main transducer of canonical TGF-β signaling. The SMAD3 mRNA level that was reduced by miR-27a-agomir could be rescued by co-transfecting with a TGF-βRI expression vector in HeLa cells (Fig. 3c). Western blot analysis detected a dramatic reduction of SMAD3 expression and phosphorylation when cells were treated with miR-27a-agomir and control vector. This reduction could be partially restored when cells were co-transfected with the TGF-βRI expression vector (Fig. 3d).

Consistent with our previous observations, dramatically decreased cell proliferation and increased apoptosis were observed in HeLa cells transfected with miR-27a-agomir and control vector; these alterations were restored by co-transfection with a TGF-βRI expression vector (Fig. 3e, g, Supplementary Fig. S5A-C). In addition, the co-transfection of miR-27a-agomir with a TGF-βRI expression vector in HeLa cells significantly restored cell migration and invasion properties (Fig. 3h, i, Supplementary Fig. S5D-E). In SiHa cells, however, the transfection of miR-27a-agomir, TGF-βRI expression vector or the combination did not affect the cell phenotype (Fig. 3e–i, Supplementary Fig. S5A-E). These findings indicate that miR-27a inhibits HeLa cell proliferation, survival, migration, and invasion by downregulating TGF-βRI.

### miR-27a suppresses cervical cancer growth in vivo

**Fig. 4:**
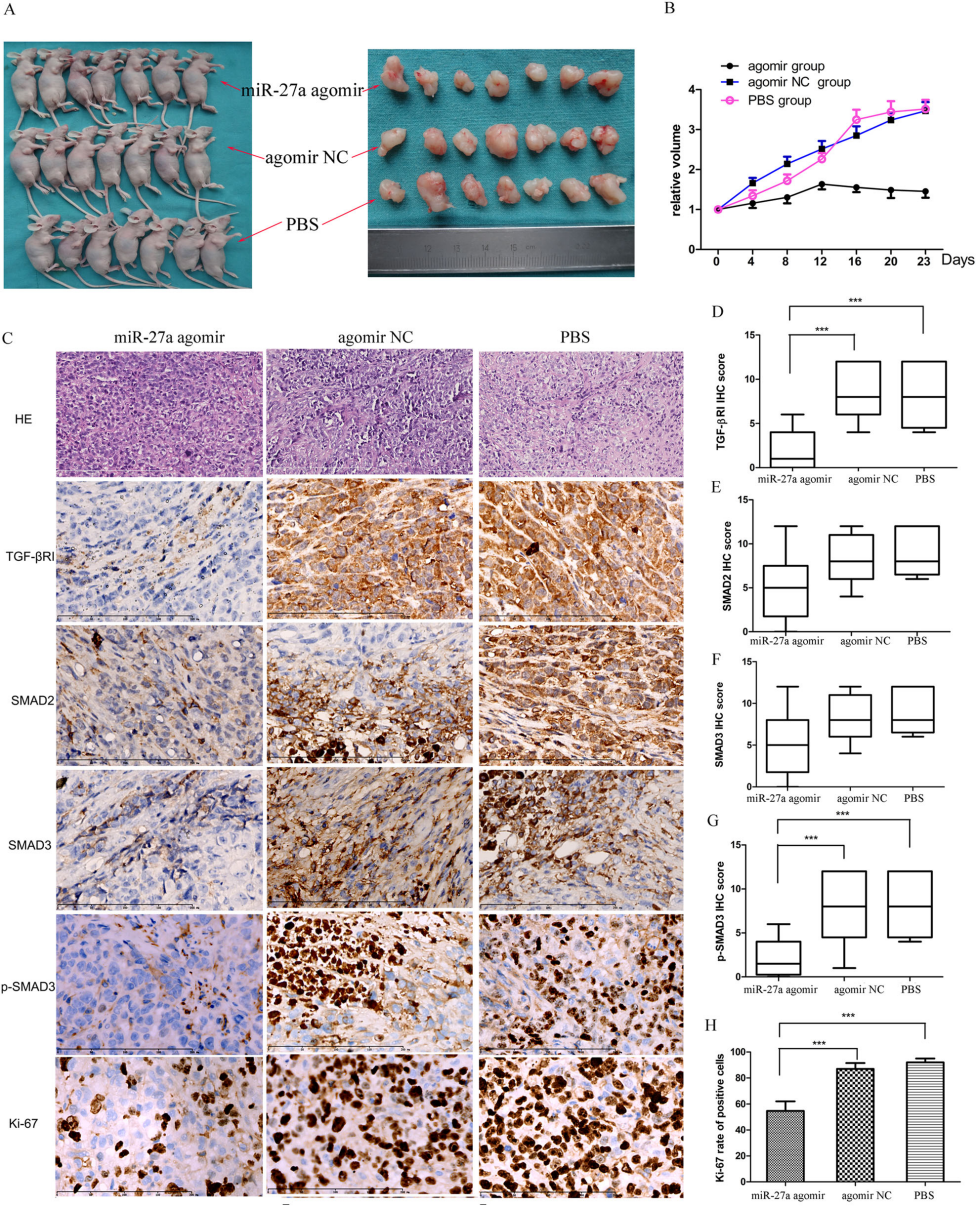
miR-27a agomir inhibits the growth of cervical cancer xenografts in vivo. Mice harboring subcutaneous tumors derived from HeLa cells received intratumoral injection of PBS, agomir NC, or miR-27a agomir (*N* = 7/group). **a** The tumor size in the agomir group is significantly smaller than the NC group and the PBS group (23 days after inoculation). **b** The curves of relative tumor volume show a significantly slower tumor growth in the agomir group compared with controls. **c** HE staining and immunohistochemical detection of TGF-βRI, SMAD2, SMAD3, p-SMAD3, and Ki-67 in the xenografts (bar, 200 µm). **d**–**h** Quantification of the immunohistochemistry results. ***P < 0.001

To further understand the tumor suppressor role of miR-27a in vivo, we subcutaneously inoculated HeLa cells into the dorsal thigh of nude mice to form xenografts. The tumor-bearing mice were randomly divided into three groups and were intratumorally injected with miR-27a-agomir, control agomir or PBS. After two weeks, the tumor size of the miR-27a-agomir group was significantly smaller compared with the control groups (Fig. 4a). Moreover, the tumor growth in the miR-27a-agomir group was significantly slower than the control groups (Fig. 4b). Immunohistochemistry analyses further uncovered significant reductions of TGF-βRI, p-SMAD3, and Ki-67 positive cells in the miR-27a agomir-injected xenografts compared with controls (Fig. 4c–h). These findings suggest that miR-27a downregulates TGF-βRI expression and inhibits tumor cell proliferation in vivo.

### miR-27a and TGF-βRI expression in human cervical cancer

**Fig. 5:**
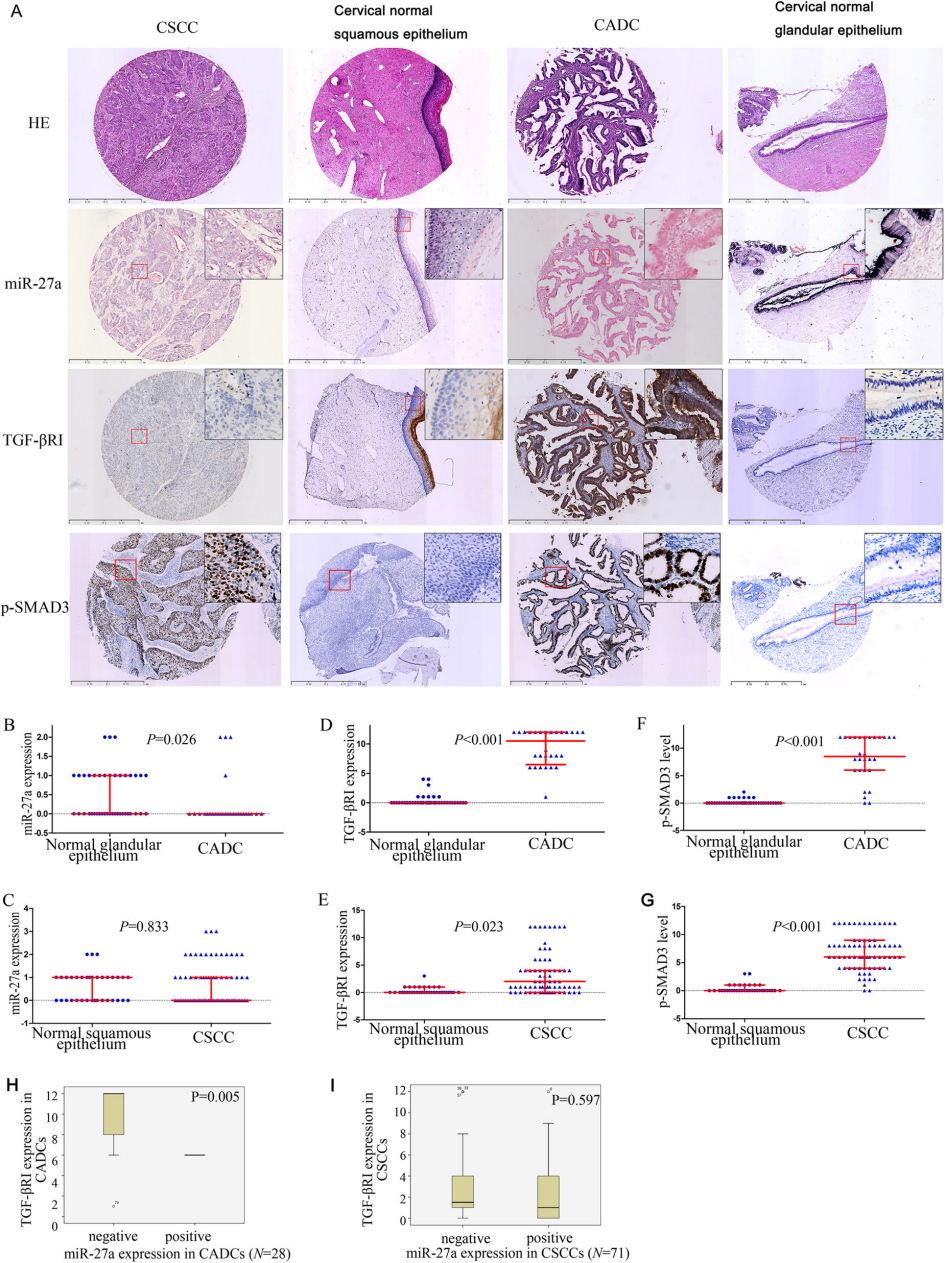
**miR-27a and TGF-βRI expression in human cervical cancer**. miR-27a was detected by tissue microarray-ISH. TGF-βRI and p-SMAD3 were detected by tissue microarray-ICH. **a** Representative images of HE staining, ISH staining for miR-27a, and IHC staining for TGF-βRI and p-SMAD3 in cervical cancers (squamous cell carcinoma, *N* = 71; adenocarcinoma, *N* = 28) and normal cervix tissues (*N* = 76). The region marked by a red rectangle is enlarged in the top-right corner of each image (400×). **b** Comparison of the miR-27a expression in cervical adenocarcinomas (CADCs) to that in normal cervix glandular epithelia. **c** Comparison of the miR-27a expression in cervical squamous cell carcinomas (CSCCs) to that in normal cervix squamous epithelia. **d** Comparison of the TGF-βRI expression in CADCs to that in normal cervix glandular epithelia. **e** Comparison of the TGF-βRI expression in CSCCs to that in normal cervix squamous epithelia. **f** Comparison of the p-SMAD3 level in CADCs to that in normal cervix glandular epithelia. **g** Comparison of the p-SMAD3 level in CSCCs to that in normal cervix squamous epithelia. **h** miR-27a expression is negatively correlated with TGF-βRI expression in CADC. **i** There is no significant correlation between miR-27a and TGF-βRI expression

Having demonstrated that miR-27a suppresses cervical cancer progression by inhibiting TGF-βRI expression and TGF-β signaling in cervical cancer, we further investigated the expression of miR-27a, TGF-βRI, SMAD2, SMAD3, and p-SMAD3 in the normal cervix (*N* = 76) and in cervical cancer tissues (*N* = 99, Fig. 5a). In situ hybridization analysis revealed a significantly reduced miR-27a expression in CADC compared with normal cervix glandular cells (*P* = 0.026, Fig. 5b), but not in squamous cell carcinoma (*P* = 0.833, Fig. 5c). Immunohistochemical staining detected a significant elevation of TGF-βRI and p-SMAD3 in both subgroups of adenocarcinoma and squamous cell carcinoma compared with normal cervix glandular epithelia and squamous epithelia, respectively (adenocarcinoma, *P*_TGF-βRI_ < 0.001, Fig. 5d, *P*_p-SMAD3_ = 0.023, Fig. 5e; squamous cell carcinoma, *P*_TGF-βRI_ < 0.001, Fig. 5f, *P*_p-SMAD3_ < 0.001, Fig. 5g). Moreover, we verified a negative correlation between miR-27a and TGF-βRI in CADCs (Pearson correlation, *P* = 0.017; Mann–Whitney *U-*test, *P* = 0.005; Fig. 5h), but not in squamous cell carcinomas (Fig. 5i).

Next, we analyzed the relationship of miR-27a and TGF-βRI to clinico-pathological characteristics in cervical cancer. The loss of miR-27a expression and high TGF-βRI expression were more frequently observed in CADCs compared with CSCCs (*P*_miR-27a_ = 0.003, 85.7 vs. 53.5%; *P*_TGF-βRI_ < 0.001, 96.4 vs. 21.1%). In addition, high TGF-βRI was significantly associated with age at diagnosis (*P* = 0.004), deep stromal invasion (*P* = 0.003), and lymph node metastasis (*P* = 0.036). There was no significant difference in miR-27a levels with respect to patients’ age, histological grade, lymphovascular space invasion, lymph node metastasis or International Federation of Gynecology and Obstetrics (FIGO) stage (Table 1). An analysis stratified according to histological type was not performed because of the limited number of CADCs (*N* = 28).

**Table 1:** The relation of miR-27a and TGF-βRI expression to clinico-pathological characteristics in human cervical cancer

Characteristics	*N*	miR-27a expression			TGF-βRI expression		
		Negative (%)	Positive (%)	*χ*^2^ test *P*	Low (%)	High (%)	*χ*^2^ test *P*
Age (year)				0.733			0.004
<45	38	23 (60.5)	15 (39.5)		23 (60.5)	15 (39.5)	
≥45	61	39 (63.9)	22 (36.1)		19 (31.1)	42 (68.9)	
Histological types				0.003			0.000
Squamous cell carcinoma	71	38 (53.5)	33 (46.5)		56 (78.9)	15 (21.1)	
Adenocarcinoma	28	24 (85.7)	4 (14.3)		1 (3.6)	27 (96.4)	
Histological grade				0.065			0.083
Well differentiated	23	19 (82.6)	4 (17.4)		9 (39.1)	14 (60.9)	
Moderately differentiated	55	30 (54.5)	25 (45.5)		33 (60.0)	22 (40.0)	
Poorly differentiated	21	13 (61.9)	8 (38.1)		15 (71.4)	6 (28.6)	
Depth of stromal invasion				0.169			0.003
<1/2	50	28 (56.0)	22 (44.0)		36 (72.0)	14 (28.0)	
≥1/2	49	34 (69.4)	15 (30.6)		21 (42.9)	28 (57.1)	
Lymphovascular space invasion			0.200			0.794	
Yes	20	15 (75.0)	5 (25.0)		11 (55.0)	9 (45.0)	
No	79	47 (59.5)	32 (40.5)		46(58.2)	33 (41.8)	
Lymph node metastasis				0.931			0.036
Yes	13	8 (61.5)	5 (38.5)		4 (30.8)	9 (69.2)	
No	86	54 (62.8)	32 (37.2)		53 (61.6)	33 (38.4)	
FIGO stage				0.121			0.161
≤IB1	73	49 (67.1)	24 (32.9)		39 (53.4)	34 (46.6)	
>IB1	26	13 (50.0)	13 (50.0)		18 (69.2)	8 (30.8)	

## Discussion

Cervical cancer remains a great threat for Chinese women’s health, with an estimated number of new cases of 98,900 and deaths of 30,500 in 2015^12^. Histologically, cervical cancer can originate from squamous epithelia (CSCC, ~80%) and glandular epithelia (CADC, ~20%). CADC represents the minority of cervical malignancies but with significantly worse prognosis than CSCC. Herein, we demonstrated that miR-27a is downregulated in cervical cancer, and elevated miR-27a antagonizes tumor progression by inhibiting TGF-βRI expression and TGF-β signaling. More importantly, low miR-27a, high TGF-βRI, and the tumor-suppressor effects of miR-27a are predominantly observed in CADCs rather than in CSCCs. These findings suggest that miR-27a and TGF-βRI may be specific biomarkers and promising therapeutic targets for cervical cancer, especially for CADC.

In the present study, we found that miR-27a expression was decreased in cervical cancer cells compared with normal cervix squamous epithelia or glandular epithelia. Moreover, miR-27a-agomir could suppress cervical cancer cell proliferation, migration, and invasion in vitro, as well as inhibit tumor growth in vivo. These findings indicate that miR-27a functions as a tumor suppressor in cervical cancer. miR-27a has also been reported to be downregulated in colorectal cancer^13,14^, oral squamous carcinoma^15^, and esophageal carcinoma^16^, in which miR-27a exhibited antitumor effects. However, in several solid tumors, miR-27a was found to be an oncogenic miRNA. High miR-27a was found in clear cell renal cell carcinomas and was identified as an independent predictor for recurrence with proliferation-promoting functions, migration-promoting functions, and invasion-promoting functions^17^. Similar results were reported in breast cancer^18^, lung adenocarcinoma^19^, non-small cell lung cancer^20^, gastric carcinoma^21^, and pancreatic carcinoma^22^. The duality of miR-27a warrants further studies of miR-27a expression and function in individual diseases.

A very recent report described an upregulated miR-27a expression in cervical cancers (*N* = 19) by qRT-PCR, and loss-of-function and gain-of-function experiments of miR-27a revealed an oncogenic role of miR-27a in HeLa and C33A cells^23^. These results are inconsistent with ours; the reasons might be as follows: the small sample size (*N* = 19) could lead to obvious selection bias; the use of qRT-PCR did not allow a precise evaluation of miR-27a expression in cervical cancer cells because the tumor tissues taken from patients usually contain other cellular components and adjacent normal tissue; only in vitro functional experiments were performed. In our study, we assessed miR-27a in a relatively large cohort of 99 cervical cancers, including 71 CSCCs and 28 CADCs, by using in situ hybridization, which allowed us to evaluate miR-27a expression, as well as the cellular and subcellular location in cervical cancer. More importantly, we compared the miR-27a expression in CSCC cells and CADC cells with normal cervical squamous cells and glandular cells, respectively. In addition, we verified the tumor suppressor role of miR-27a in animal models.

We identified TGF-βRI as a direct target of miR-27a, which contributed to the tumor suppressor role of miR-27a in cervical cancer. TGF-βRI is a membrane serine/threonine protein kinase receptor that forms a hetero-complex with TGF-βRII upon TGF-β stimulation, mediating the phosphorylation of SMAD proteins and subsequent TGF-β signaling^24^. The role of the TGF-β signaling pathway in tumors is context-dependent, as it can function as both a suppressor and a promoter of tumor progression and invasion^24^. In cervical cancer, TGF-β acts as a tumor suppressor in malignant transformation, but in the early stage of the disease, it promotes tumor progression by promoting metastatic properties, epithelial-mesenchymal transition, angiogenesis, and escape from immune surveillance. This role-switch from a tumor suppressor to a tumor promoter of TGF-β is considered to be partially attributed to mutations and loss of expression of TGF-βR and SMAD proteins^25^. We verified that miR-27a downregulates TGF-βRI mRNA by binding to its 3′-UTR. At the same time, we showed a generally suppressed transcription of SMADs in cells treated with miR-27a agomir. Indeed, miR-27a has been identified as a basal transcription regulator by negatively regulating the p44 subunit of general transcription factor IIH through direct interaction with the 3′-UTR of p44 mRNA^26^. Thus, miR-27a inhibits the TGF-β pathway through targeting multiple factors involving TGF-βRI and SMAD proteins via direct and indirect interactions, thereby favoring tumor progression.

High-risk HPV infection plays a crucial role in the etiology of cervical cancer. The most oncogenic HPV subtypes are HPV16 and HPV18; they are associated with ~70% of invasive cervical cancers^27^. Wang X, et al. reported that thirteen abundant host miRNAs, including miR-27a, were specifically regulated by HPV16 and HPV18, as determined by miRNA array in combination with small RNA sequencing; the decrease of miR-27a could be attributed to viral oncoproteins E6 and E7. Most CSCCs are HPV16-positive, whereas CADCs harbor predominantly HPV18 that is associated with an unfavorable prognosis^28^. Intriguingly, we found dramatically reduced expression and tumor-suppressor function of miR-27a in human CADC tissue specimens and a CADC-derived cell line (HPV18-positive HeLa), but not in CSCC tissue and CSCC-derived cell lines (HPV16-positive SiHa and CaSki). Although we found low miR-27a levels in both adenocarcinoma cell lines HeLa and C33A, the function of miR27a as a tumor suppressor was only observed in HeLa, but not in C33A. HeLa is a CADC of usual type derived from HPV18-infection. On the other hand, C33A is HPV-negative adenocarcinoma with p53 mutation (PMID:10410876), which mimics the genetic background of gastric subtype. Clinical features are quite different between usual type and gastric type. Given varied pathological types, HPV status and genetic characteristics, it is not surprising that they do not share the same mechanism regarding miR-27a.

A recent study reported that TGF-βRI functions as a key regulator during lineage commitment and differentiation of lung cancer cells^29^. The expression of a constitutively active TGF-βR1 facilitates the formation of adenocarcinoma, whereas repression of the TGF-β pathway at the receptor level promotes the formation of squamous cell carcinoma^29^. These findings indicate the possibility that HPV18 might regulate the lineage commitment and differentiation of CADCs by manipulating miR-27a expression and the subsequent inhibition of TGF-βR1 and TGF-β signaling pathway. However, further evidence is required to verify this hypothesis.

Although CADC is less frequent than CSCC, CADC patients have a significantly poorer survival than CSCC patients^28^. Hence, it is of great clinical significance to clarify the molecular mechanisms underlying CADC progression. Our findings suggest that miR-27a inhibits TGF-β signaling pathway in cervical cancer, which in turn suppresses tumor progression. This mechanism is likely specific for CADC rather than CSCC, because CSCC had a higher miR-27a level than CADC, the malignant properties of CSCC cells were not affected by miR-27a agomir in vitro, and a significant correlation between miR-27a and TGF-βRI was not found in CSCC. In addition, our findings underline the role of miR-27a linking HPV infection to TGF-β signaling in CADC. Given the critical role of TGF-β pathway in cervical cancer progression, these findings may lead to novel treatment strategies for CADC.

## Materials and methods

### Cell culture, transfection, and treatment

HeLa (CADC of usual type, HPV 18 positive), SiHa CSCC, C33A (non-HPV associated CADC of gastric type with P53 mutation, PMID:10410876), and CaSki CSCC, derived from small intestine metastasis) cell lines were purchased from American Type Culture Collection. The cell lines were re-authenticated at Shanghai Biowing Applied Biotechnology Co. Ltd (China) via STR (short tandem repeat) profiling. Cells were cultured in DMEM/F12 medium containing 10% fetal bovine serum (GIBCO, USA) at 37 °C with 5% CO_2_. Normal human cervical epithelium cells were cervical exfoliated cells taken from women without intraepithelial lesions or malignancy, which was confirmed by a liquid-based cytology test (LCT, BDSure Path™, USA); the cells were stored in nuclease-free PBS at 4 °C for RNA isolation within 24 h.

A miR-27a-agomir and a TGF-βRI expression vector were used to enhance miR-27a function and TGF-βRI expression, respectively. Agomirs are chemically-modified double-strand miRNA mimics, which can efficiently mimic mature endogenous miRNAs after transfection into cells. Transfection of miR-27a-agomir and miR-27a-antagomirs, and the TGF-βRI expression vector was carried out using Lipofectamine 2000 (Invitrogen, USA) according to the manufacturer’s protocols. Hsa-miR-27a-agomir, miR-27a-antagomir 5p, miR-27a-antagomir 3p, and their scrambled negative controls were designed and synthesized by GenePharma (Shanghai, China); their sequences are listed in Supplementary Table 1. The TGF-βRI expression vector Myc-DDK-hTGF-βRI and the control vector PCMV6-Entry were purchased from OriGene (Maryland, USA). The TGF-βRI kinase activity inhibitor A83-01 was purchased from Tocris Bioscience (Bristol, UK), and the cells were treated with a final concentration of 10 μM for 72 h^30^.

### Expression analysis

Total RNA from cells was purified using Trizol (Invitrogen, USA) and reverse transcribed using a TaqMan kit (ABI, USA). The expression of miRNAs was determined using TaqMan microRNA Assay kits (ABI, USA) with U6 as a reference. The expression levels of TGF-βRI/II and SMADs were assayed by quantitative real-time PCR (qRT-PCR) using SYBR Green PCR Master Mix (Toyobo, Japan) with β-actin as a reference. The relative miRNA and mRNA levels were calculated by the standard 2^−ΔΔCt^ method. The primers used are listed in Supplementary Table S1.

### Cell proliferation, apoptosis, migration, and invasion assays

The cell proliferation activity was assessed by EdU (5-ethynyl-2′-deoxyuridine) incorporation and flow cytometry. The EdU assay was performed according to the manufacturer′s instructions (RiboBio, Guangzhou, China), and the percentage of EdU-positive cells was used to evaluate cell proliferation. For flow cytometry, the cells were stained with 2 µM CFSE (Sigma, USA). Data were analyzed using Cell Quest and ModFitLT software (BD, USA), and the proliferation index was calculated as previously described^31^. For apoptosis, the cells were assayed using a FITC AnnexinV Apoptosis Detection Kit (BD, USA) followed by flow cytometry analysis. For in vitro transwell assays, 1 × 10^5^ cells were seeded in the upper chamber of a culture insert (8-mm pore size in 24-well plates, BD, USA) without (migration assay) or with (invasion assay) Matrigel (BD, USA) in serum-free media. The bottom chamber was filled with the same medium supplemented with 10% fetal bovine serum. After 24 h, the cells on the undersurface of the membrane were stained with crystal violet and counted using a light microscope. The cell migration and invasion properties were evaluated by the number of cells per high power field. All experiments were performed in triplicate and with at least three biological replicates.

### Luciferase reporter assay

The 3′-UTR of TGFβ-RI mRNA (F:GCGGCTCGAGGAGGTGGTAGCTAAAGAACA, R:AATGCGGCCGCTAATGACTGAAGGAAATGGA) and TGFBRI-mut mRNA(F:CTTTTATCTGACACTTCTTATTCTGAGGGGAGA, R:GAATAAGAAGTGTCAGATAAAAGGACTTCGAAA) were fused to the 3′-end of the luciferase gene in the pmiR-RB-REPORT™ vector (Riobobio, Guangzhou, China). The vectors were transfected into 293 T cells in the absence or presence of hsa-miR-27a-3p mimics. The luciferase reporter assays were carried out according to the manufacturer’s protocol.

### Mouse xenograft models

All mice were manipulated and housed according to protocols approved by the Tongji Medical College’s Animal Care and Use Committee. To induce tumors, 2 × 10^6^ HeLa cells were inoculated subcutaneously into the right axilla of age-matched BALB/c-nu/nu female nude mice (3–4 weeks of age). Once the tumor volume reached 100 mm^3^, the mice were randomly divided into three groups (*n* = 7/group) and injected intratumorally with 2 nmol miR-27a-agomir, agomir-NC, or PBS twice a week for two weeks. The tumor size was measured with a caliper every three days using the following formula: volume = length × (square of width)/2. One week after the last injection, the mice were sacrificed by cervical dislocation under anesthesia. The tumor tissues were paraffin-embedded and sliced. Hematoxylin-eosin (HE) staining and immunohistochemistry staining with antibodies against TGF-βRI (GeneTex, USA), SMAD3 (CST, USA), p-SMAD3 (Abcam, USA), and Ki67 (Abcam, USA) were performed. The percentage of Ki-67 positive cells was derived by averaging five randomly selected fields of view. Immunohistochemistry scores were evaluated and recorded by two pathologists at the four linear score levels (0–4) according to the percentage and the color of positive cells on each slide. The final immunohistochemistry score was the multiplication of scores from each pathologist.

### Human samples and tissue microarray

The protocol for the human study was reviewed and approved by the Research Ethics Board in Wuhan Union Hospital (Wuhan, China), and informed consent was obtained from all the patients. The cervical tissues were derived from surgically removed tissues of inpatients in Wuhan Union Hospital, Tongji Medical College, Huazhong University of Science and Technology (Wuhan, China) from Jan. 2014 to Dec. 2014. In total, 99 patients with invasive cervical cancer were enrolled. All patients did not receive radiotherapy or chemotherapy prior to surgery, did not have other malignancies within five years before cervical cancer diagnosis, and diagnoses were pathologically confirmed. Among them, 71 patients were diagnosed with CSCC, and 28 patients diagnosed with CADC. According to the International FIGO, 73 cases were in stage IA-IB1, and 26 cases were in stage IB2-IIA2. Normal cervical tissues (*N* = 76) derived from patients who received total hysterectomy due to uterine benign lesions were used as the control samples. Among them, 35 and 41 cases served as normal controls for CSCC and CADC, respectively, according to the presence of normal squamous cells or glandular cells. The average age of the cervical cancer group and the control group was 48 years (range, 29–76 years) and 44 years (range, 36–76 years), respectively. For the tissue microarray, archival paraffin-embedded tissue blocks were used. The most representative tumor areas (neoplastic cells) were carefully selected, marked on the HE slide and sampled. From each specimen, tissue cores with a diameter of 2 mm were punched and then arrayed on a recipient paraffin block. The microarray blocks were designed by tissue microarray designer tissue array design software (ALPHELYS SARL, France), constructed with MiniCore Control Station (ALPHELYS SARL, France), and assembled using a tissue-arraying instrument (Beecher Instruments)^32^. Multiple 4-μm sections were cut with a micron microtome (HM355S) for subsequent immunohistochemistry analysis, and 6-μm sections were used for the in situ hybridization of miR-27a.

### In situ hybridization

The miR-27a in paraffin-embedded normal cervix and cervical cancer tissues was detected by in situ hybridization. The miRCURY LNA^TM^ Detection probes for miR-27a (hsa-miR-27a) (Exiqon, Denmark) were used. The sequences (enhanced with LNA) were 5′-GCGGAACTTAGCCACTGTGAA-3′-labeled with a digoxigenin (DIG) at both the 5′-ends and 3′-ends. The U6 (hsa/mmu/rno) and scrambled miRNA were used as internal and negative controls, respectively. In situ hybridization was performed according to the manufacturer’s protocol (Exiqon, Denmark), and the results were evaluated, scored, and recorded independently by two pathologists. The number of positive-staining cells in ten high-power microscopic fields was counted, and the percentage of positive cells was calculated. The percentage of positive cells was assessed semi-quantitatively as 0 (0–9%) or 1 (≥10%). The staining intensity was categorized as 0 (negative), 1 (weak), 2 (moderate) or 3 (strong). These two scores were multiplied, and the final in situ hybridization score was determined (value 0–3).

### Statistical analysis

Statistical analyses were performed with SPSS 17.0 software. The numeric data of the in vitro experiments are expressed as the mean ± standard deviation, and the differences between groups were assessed by one-way analysis of variance and multiple comparisons. The differences in the expression of miR-27a, TGF-βRI, SMAD3, and p-SMAD3 were assessed with the Mann–Whitney U test or Kruskal–Wallis test, as appropriate. In the analysis of the correlation between the expression of miR-27a and TGF-βRI and the clinico-pathological parameters of cervical cancer, the miR-27a expression was divided into two categories of “negative” (0) and “positive” (≥1); the TGF-βRI expression was categorized as “low” (<6) and “high” (≥6). The correlations were tested using the *χ*^2^ test. All tests were two-tailed, and a value of *P* < 0.05 was considered statistically significant.

## Electronic supplementary material

Supplemental figure S1(TIF 5775 kb)

Supplemental figure S2(TIF 4416 kb)

Supplemental figure S3(TIF 2261 kb)

Supplemental figure S4(TIF 349 kb)

Supplemental figure S5(TIF 16439 kb)

Supplemental figure legends(DOCX 14 kb)

Supplemental table S1(DOCX 18 kb)
