# Supplementary figures and images for: miR-27a inhibits cervical adenocarcinoma progression by downregulating the TGF-βRI signaling pathway
Source: Cell Death Dis. 2018 Mar 12;9(3):395. doi: 10.1038/s41419-018-0431-2 (PMC5847584; doi:10.1038/s41419-018-0431-2)

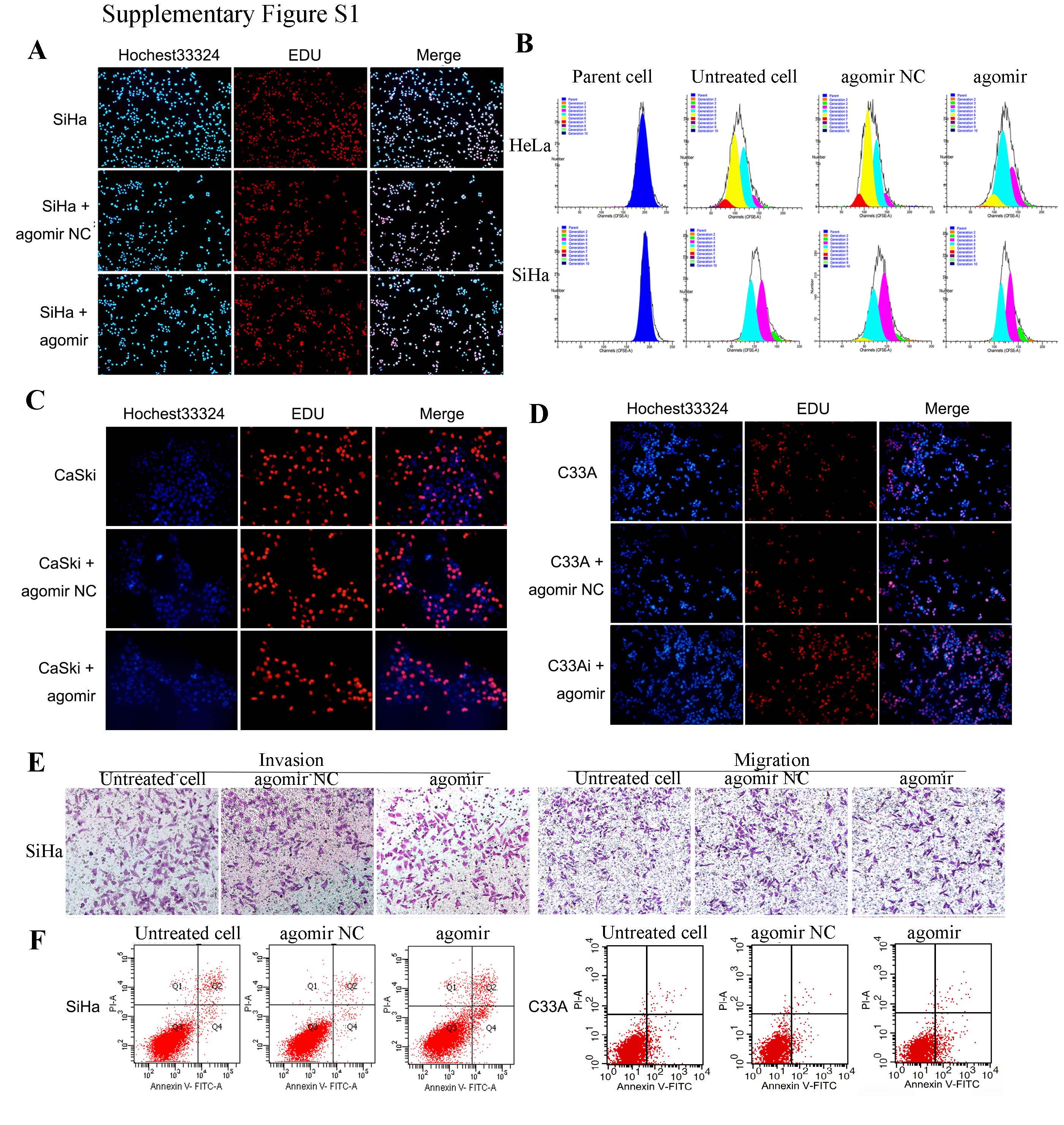

Supplement: Supplementary file 1 — Supplemental figure S1(TIF 5775 kb) [file 41419_2018_431_MOESM1_ESM.tif]

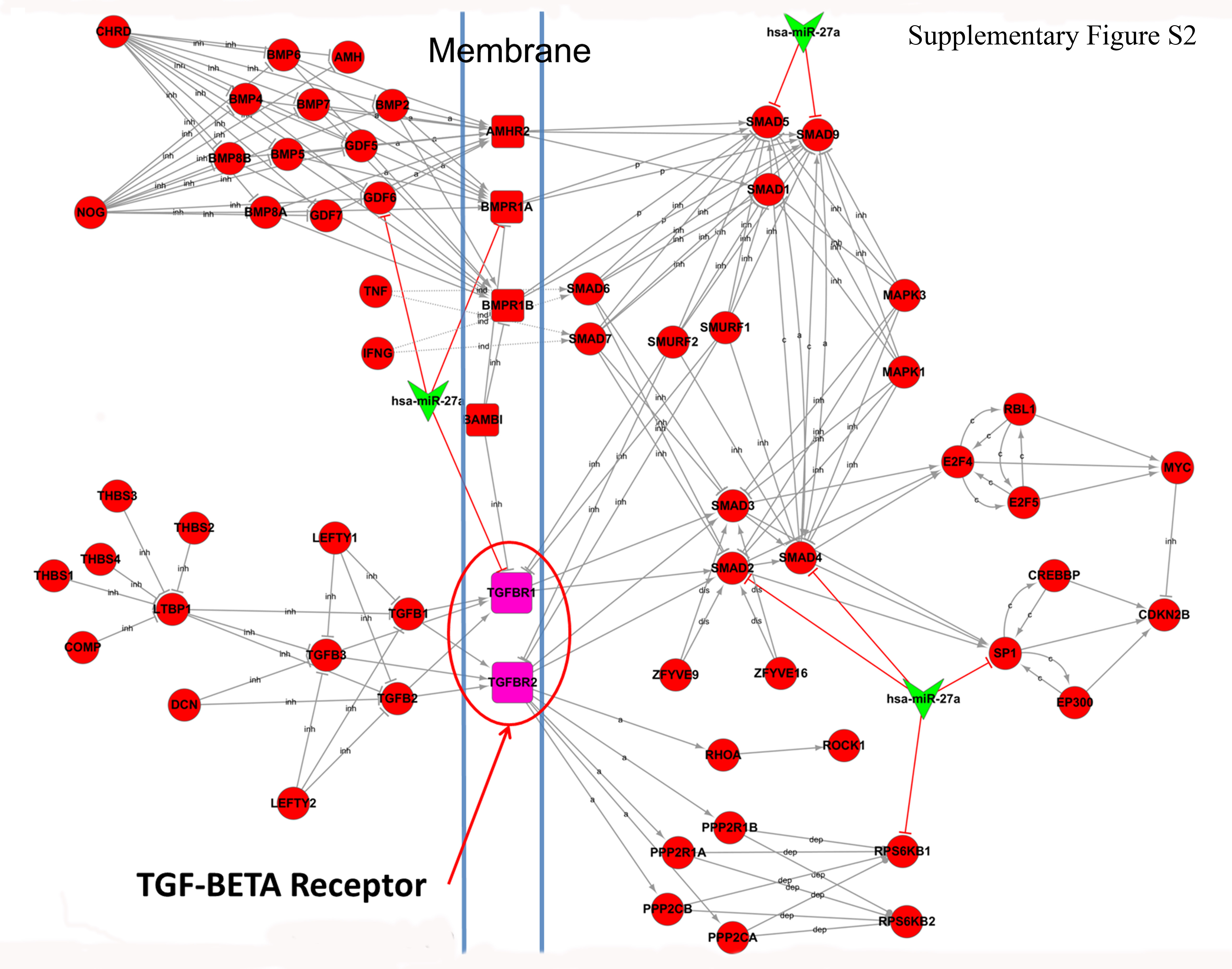

Supplement: Supplementary file 2 — Supplemental figure S2(TIF 4416 kb) [file 41419_2018_431_MOESM2_ESM.tif]

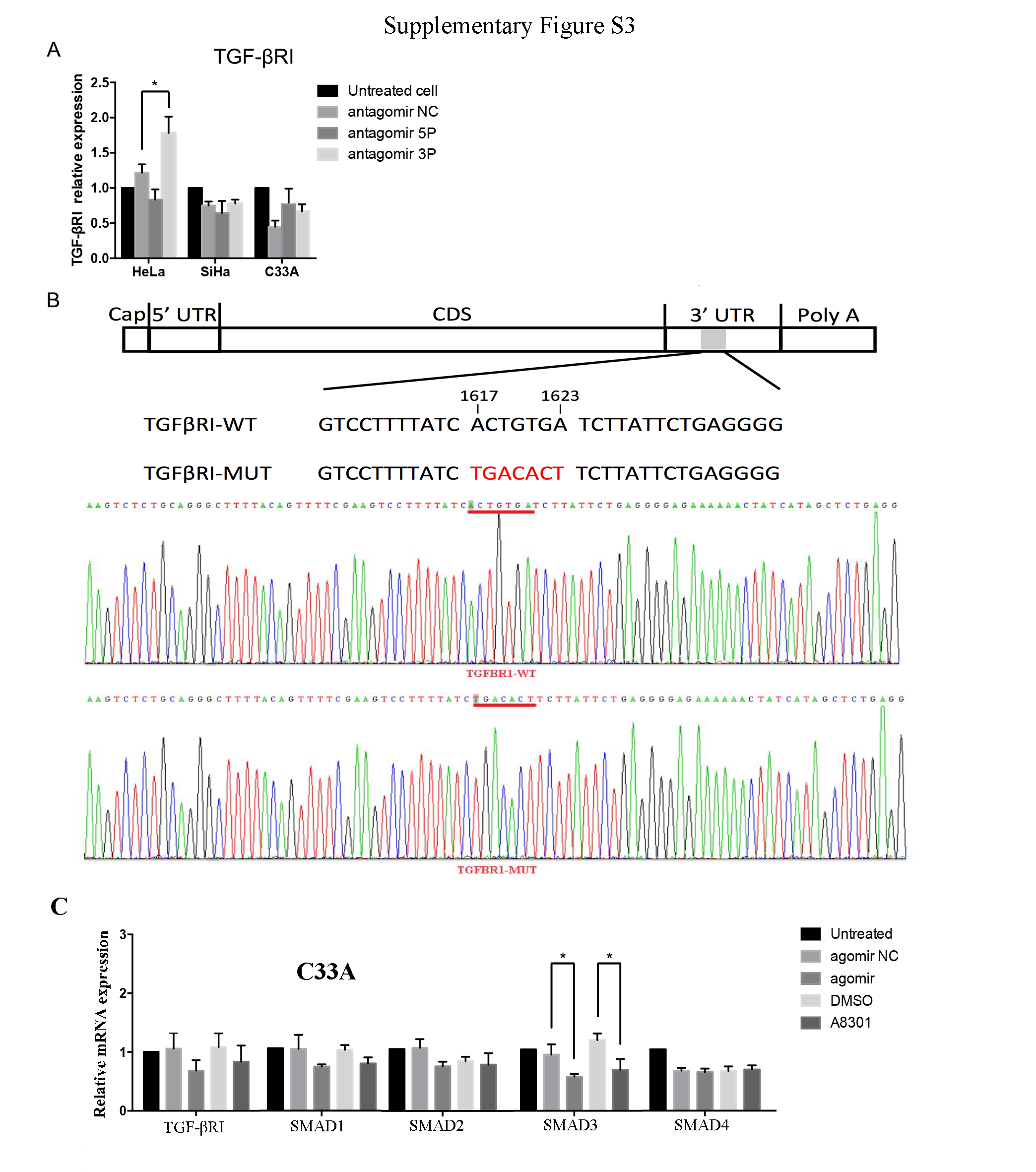

Supplement: Supplementary file 3 — Supplemental figure S3(TIF 2261 kb) [file 41419_2018_431_MOESM3_ESM.tif]

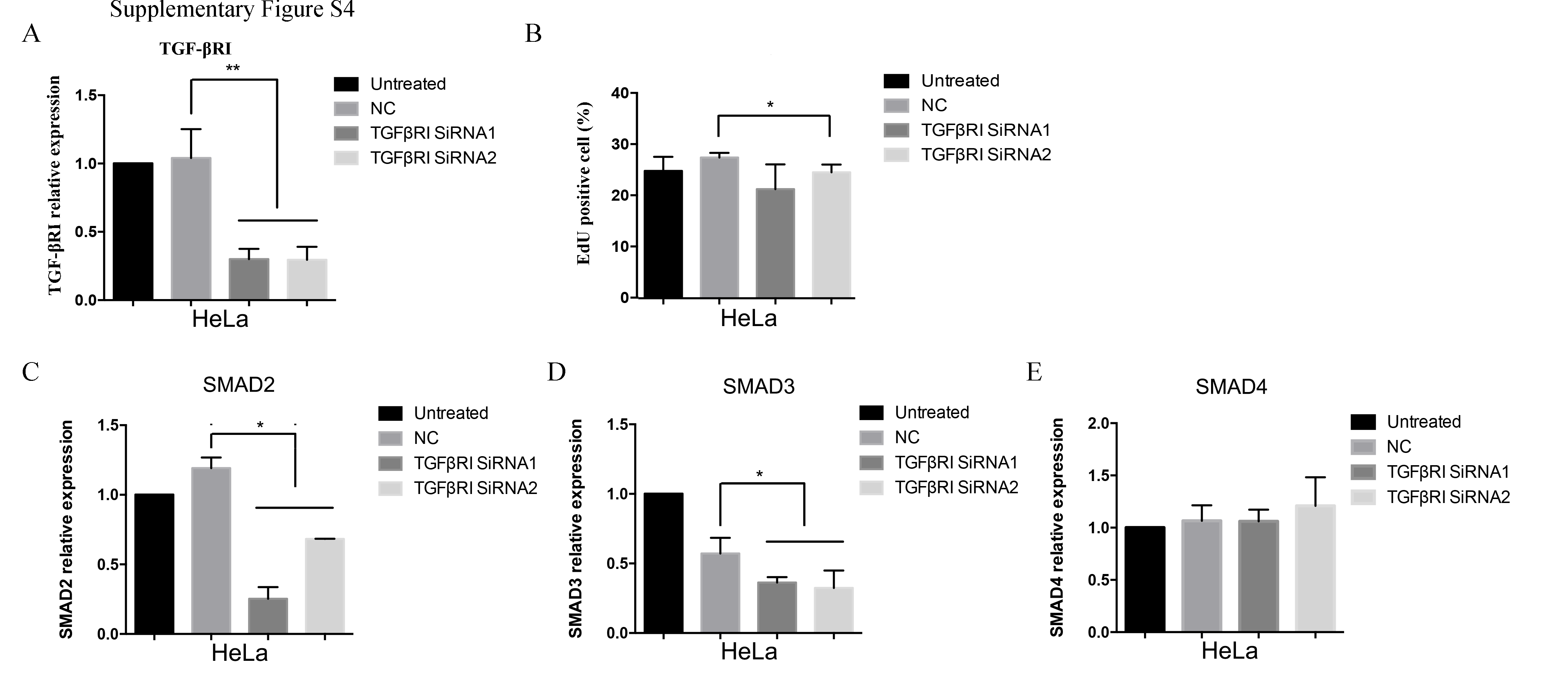

Supplement: Supplementary file 4 — Supplemental figure S4(TIF 349 kb) [file 41419_2018_431_MOESM4_ESM.tif]

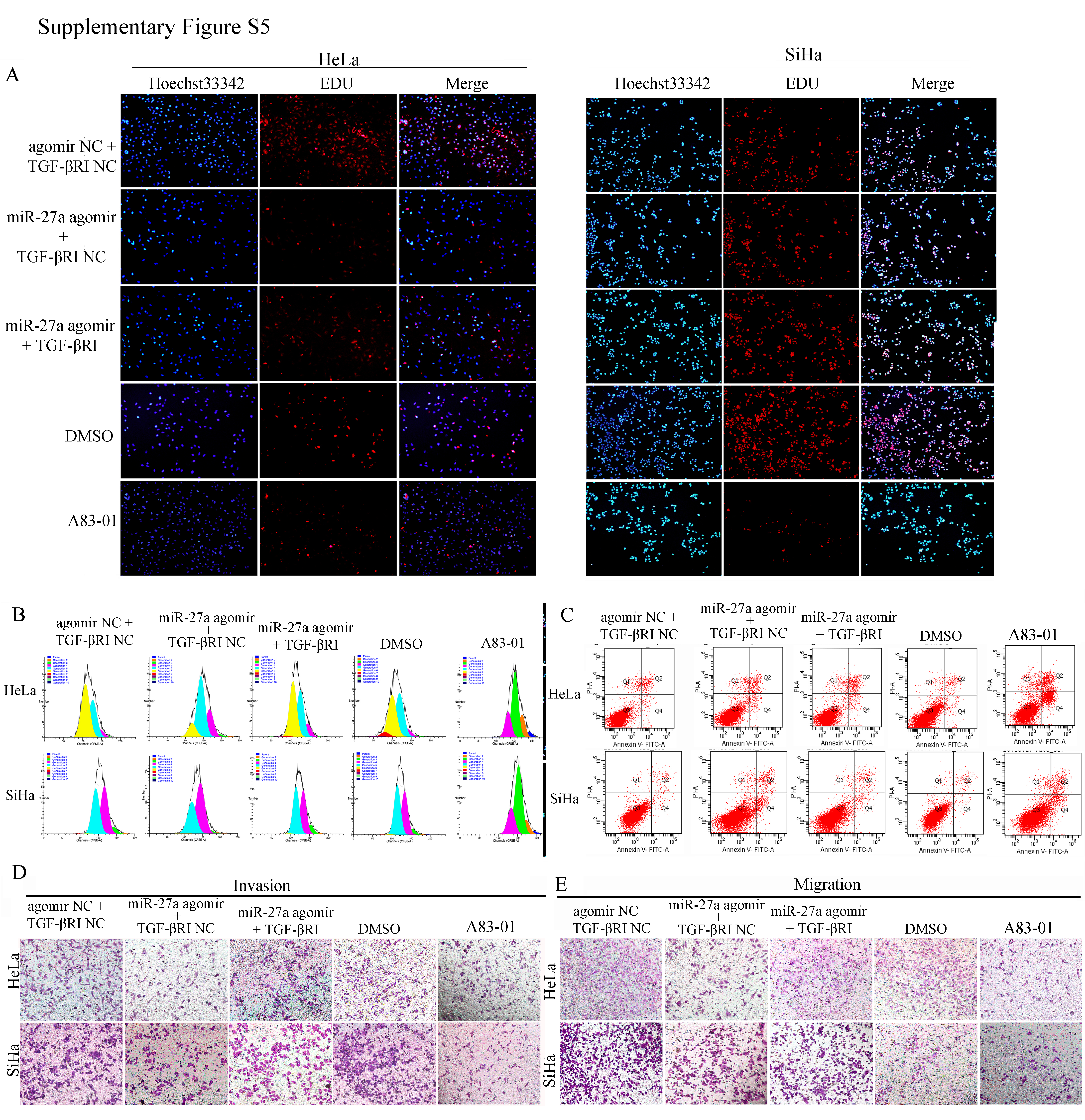

Supplement: Supplementary file 5 — Supplemental figure S5(TIF 16439 kb) [file 41419_2018_431_MOESM5_ESM.tif]
