# Supplementary material for: miR-27a inhibits cervical adenocarcinoma progression by downregulating the TGF-βRI signaling pathway
Source: Cell Death Dis. 2018 Mar 12;9(3):395. doi: 10.1038/s41419-018-0431-2 (PMC5847584; doi:10.1038/s41419-018-0431-2)
Supplement: Supplementary file 6 — Supplemental figure legends(DOCX 14 kb) [file 41419_2018_431_MOESM6_ESM.docx]

Supplemental figure legends

**Figure S1** Cells were treated with miR-27a agomir and cell proliferation, invasion, migration, and apoptosis were assessed. (A) Representative images of EdU incorporation assays of SiHa cells. Proliferating nucleus were labeled with EdU (red) while total nuclei were counterstained with Hoechst33342 (blue). (B) Representative images of flow cytometry analysis of CFSE-positive HeLa and SiHa cells show the cell fractions of sequential generations (depicted by the different colors). (C) Representative images of EdU incorporation assays of CaSki cells. (D) Representative images of EdU incorporation assays of C33A cells. (E) Representative images of invasion and migration assays of SiHa cells. (F) Representative images of flow cytometry apoptosis assays of SiHa cells.

**Figure S2** GeneGO analysis revealed that TGF-βRI was a potential target of miR-27a.

**Figure S3** miR-27a targets TGF-βRI. (A) Cells were treated with miR-27a antagomirs. TGF-βRI mRNA levels were assessed by qRT-PCR. β-actin served as internal control. (B) The schematic diagram shows the predicted miR-27a binding sites in the 3'-UTR of TGF-βRI mRNA and the mutations we preformed. The results of sequencing analysis are shown. (C) qRT-PCR assays for mRNA levels of TGF-βRI and SMADs in C33A cells transfected with miR-27a agomir versus negative control and those treated with A8301 versus DMSO.

**Figure S4** Effects of TGF-βRI depletion on cell proliferation and expression of SMADs in HeLa cells. (A) qRT-PCR. SiRNAs significantly reduced TGF-βRI mRNA levels in HeLa cells. (B) The percent of EdU-positive cells was decreased after transfection of a TGF-βRI siRNA. (C) qRT-PCR. TGF-βRI siRNAs reduced SMAD2 mRMA expression. (D) qRT-PCR. TGF-βRI siRNAs reduced SMAD3 mRMA expression. (E) qRT-PCR. SMAD4 expression was not significantly affected by qRT-PCR.

**Figure S5** miR-27a functions as a tumor suppressor through repressing TGF-βRI expression and TGF-β signaling. Cells were transfected with miR-27a agomir in combination with TGF-βRI expression vector or control vector. (A) Representative images of EdU incorporation assays of HeLa and SiHa cells. (B) Representative images of flow cytometry analysis of CFSE-positive HeLa and SiHa cells. (C) Representative images of flow cytometry apoptosis assays of HeLa and SiHa cells. (D) Representative images of transwell invasion assays of HeLa and SiHa cells. (E) Representative images of transwell migration assays of HeLa and SiHa cells.
