# Supplementary material for: miR-27a inhibits cervical adenocarcinoma progression by downregulating the TGF-βRI signaling pathway
Source: Cell Death Dis. 2018 Mar 12;9(3):395. doi: 10.1038/s41419-018-0431-2 (PMC5847584; doi:10.1038/s41419-018-0431-2)
Supplement: Supplementary file 7 — Supplemental table S1(DOCX 18 kb) [file 41419_2018_431_MOESM7_ESM.docx]

Supplementary Table 1  Sequences of nucleotides used

| Name | Sequences |
| --- | --- |
| hsa-miR-27a-agomir (Forward) | 5’-UUCACAGUGGCUAAGUUCCGC-3’ |
| hsa-miR-27a-agomir (Reverse) | 5’-GGAACUUAGCCACUGUGAAUU-3’ |
| hsa-miR-27a-agomir-NC (Forward) | 5’-GCGACGAUCUGCCUAAGAU-3’ |
| hsa-miR-27a-agomir-NC (Reverse) | 5’-AUCUUAGGCAGAUCGUCGC-3’ |
| hsa-miR-27a-5p-antagomir | 5’-AGGGCTTAGCTGCTTGTGAGCA-3’ |
| hsa-miR-27a-3p-antagomir | 5’-TTCACAGTGGCTAAGTTCCGC-3’ |
| β-actin (Forward) | 5'-GCCAACACAGTGCTGTCTGG-3' |
| β-actin (Reverse) | 5'-GCTCAGGAGGAGCAATGATCTTG-3' |
| TGF-βRI (Forward) | 5’-GTTAAGGCCAAATATCCCAAACA-3’ |
| TGF-βRI (Reverse) | 5’-ATAATTTTAGCCATTACTCTCAAGG-3’ |
| TGF-βRII (Forward) | 5’-GGCCGCTGCACATCGT-3’ |
| TGF-βRII (Reverse) | 5’-CATTTCCACATCCGACTTCTGA-3’ |
| SMAD1(Forward) | 5’-CTCATGTCATTTACTGCCGTGT-3’ |
| SMAD1(Reverse) | 5’-TATTCGCTGTGTCTTGGAACC-3’ |
| SMAD2 (Forward) | 5’-GCCTTTACAGCTTCTCTGAACAA-3’ |
| SMAD2 (Reverse) | 5’-ATGTGGCAATCCTTTTCGAT-3’ |
| SMAD3 (Forward) | 5’-CCCCAGCACATAATAACTTGG-3’ |
| SMAD3 (Reverse) | 5’-AGGAGATGGAGCACCAGAAG-3’ |
| SMAD4 (Forward) | 5’-GTCAAGATCTTCAGGCATGGCTCAGAGCTT-3’ |
| SMAD4 (Reverse) | 5’-GTCAAAGCTTTTACTCTTGGTAAAATTAACTCACCCACA-3’ |
| SMAD5 (Forward) | 5’-GGCTTTCATCCCACCACTGTC-3’ |
| SMAD5 (Reverse) | 5’-TATGGGGTTCAGAGGGGAGCC-3’ |
| SMAD8 (Forward) | 5’-CAGGCTTCCTCCCGAAGTG-3’ |
| SMAD8 (Reverse) | 5’-ACATTAGAAAGAAGTCCAAGACAGAATCT-3’ |
